# Gender Disparities in Invited Commentary Authorship in 2459 Medical Journals

**DOI:** 10.1001/jamanetworkopen.2019.13682

**Published:** 2019-10-23

**Authors:** Emma G. Thomas, Bamini Jayabalasingham, Tom Collins, Jeroen Geertzen, Chinh Bui, Francesca Dominici

**Affiliations:** 1 Harvard T.H. Chan School of Public Health, Harvard University, Boston, Massachusetts; 2 Elsevier Inc, New York, New York; 3 Elsevier BV, Amsterdam, the Netherlands; 4 Harvard Data Science Initiative, Harvard University, Cambridge, Massachusetts

## Abstract

**Importance:**

In peer-reviewed medical journals, authoring an invited commentary on an original article is a recognition of expertise. It has been documented that women author fewer invited publications than men do. However, it is unknown whether this disparity is due to gender differences in characteristics that are associated with invitations, such as field of expertise, seniority, and scientific output.

**Objective:**

To estimate the odds ratio (OR) of authoring an invited commentary for women compared with men who had similar expertise, seniority, and publication metrics.

**Design, Setting, and Participants:**

This matched case-control study included all medical invited commentaries published from January 1, 2013, through December 31, 2017, in English-language medical journals and multidisciplinary journals. Invited commentaries were defined as publications that cite another publication within the same journal volume and issue. Bibliometric data were obtained from Scopus. Cases were defined as corresponding authors of invited commentaries in a given journal during the study period. Controls were matched to cases based on scientific expertise by calculating a similarity index for abstracts published during the same period using natural language processing. Data analyses were conducted from March 13, 2019, through May 3, 2019.

**Exposure:**

Corresponding or sole author gender was predicted from author first name and country of origin using genderize.io.

**Main Outcomes and Measures:**

The OR for gender was estimated after adjusting for field of expertise, publication output, citation impact, and years active (ie, years since first publication), with an interaction between gender and years active.

**Results:**

The final data set included 43 235 cases across 2549 journals; there were 34 047 unique intraciting commentary authors, among whom 9072 (26.6%) were women. For researchers who had been active for the median of 19 years, the odds of invited commentary authorship were 21% lower for women (OR, 0.79 [95% CI, 0.77-0.81]; *P* < .001) compared with men who had similar scientific expertise, number of publications, and citation impact. For every decile increase in years active, the OR decreased by a factor of 0.97 (95% CI, 0.96-0.98; *P* < .001).

**Conclusions and Relevance:**

In this case-control study, women had lower odds of authoring invited commentaries than their male peers. This disparity was larger for senior researchers. Journal editors could use natural language processing of published research to widen and diversify the pool of experts considered for commentary invitations.

## Key Points

**Question:**

Is gender associated with authorship of invited commentaries in medical journals among authors with comparable scientific credentials?

**Findings:**

In this case-control study of invited commentaries published in 2459 journals from January 1, 2013, through December 31, 2017, the odds of authoring an invited commentary were 21% lower for women compared with men who had similar fields of expertise and publication metrics among researchers who had been actively publishing for the median of 19 years.

**Meaning:**

Women had lower odds of authoring invited commentaries in medical journals compared with men with similar scientific expertise, seniority, and publication metrics.

## Teaser

This case-control study examines gender differences in authorship of invited commentaries published in medical journals from 2013 to 2017, controlling for field of expertise, seniority, and publication metrics.

## Introduction

Women are underrepresented as authors of peer-reviewed publications across a wide variety of scientific fields,^1,2^ including many areas of medical research^3,4,5,6,7,8^ and nonresearch medical publications.^9^ According to a 2018 study by Holman et al,^10^ most disciplines are years or decades from achieving gender parity. The gender gap is widest at top-tier journals and for prestigious authorship positions.^10,11,12,13,14^ Bibliometric analyses suggest that women review papers less frequently than male peers,^15^ are cited less often,^11,12,13,16^ and are underrepresented on editorial boards.^13,17,18^ Single-journal analyses of acceptance rates after peer review have often found no evidence for gender differences,^19,20,21,22,23,24^ although imbalances favoring men have been observed in larger data sets.^25,26^

In medical journals, publications that have been solicited by the editors are a recognition of expertise and can raise the author’s profile. Common types of solicited publications include commentaries, editorials, and reviews. The proportion of women authoring invited articles is low^22,25,27,28,29,30^ even when compared with the proportion of female authors of all articles.^10^ The probability of authoring an invited publication depends on scientific field, seniority, and publication record, factors that are also associated with gender^10,31^; these associations may explain the disparity in solicited publication authorship. However, to our knowledge, previous studies of gender and invited article authorship did not control for these factors.^10,22,25,27,28,29,30^ This raises the question of whether women author fewer invited articles than men with similar scientific expertise and credentials.

In this case-control study, we estimated the association of gender with authorship of medical invited commentary articles in peer-reviewed medical and interdisciplinary journals, controlling for field of expertise, author seniority, and publication metrics. This was not a descriptive analysis of gender disparities; we investigated whether gender differences in invited commentary authorship were reduced or eliminated after adjustment for key author characteristics. We obtained journal-specific and pooled estimates and investigated modifiers of the association of gender with invited commentary authorship, including author seniority, journal topic, and journal impact.

## Methods

This study did not meet the definition of human subjects research under US federal regulation 45 CFR§46.102(e); therefore, institutional review board oversight was not required. We observed the Strengthening the Reporting of Observational Studies in Epidemiology (STROBE) reporting guidelines for case-control studies.

### Data

We extracted bibliometric data from Scopus,^32^ a comprehensive online database of peer-reviewed abstracts and citations. Scopus has been shown to be a reliable source for studying life sciences publication patterns.^33^

### Outcome

The outcome was corresponding authorship on an invited commentary article. To identify invited commentaries, we searched for intraciting commentary (ICC) articles, defined as publications that cite 1 or more other publications (the focal article) in the same journal volume and issue. Articles with missing volume or issue number were excluded. We restricted our analysis to ICCs for 3 reasons: (1) ICCs are a common type of invited commentary in medical journals; (2) ICCs are almost certainly solicited by journal editors, since citing another publication in the same journal volume and issue requires advance knowledge of the focal article and its publication timeline; and (3) ICCs can be identified through automated search in Scopus. Although other types of solicited publications exist (eg, invited reviews, opinion articles), no consistent scheme exists for identifying these publications across journals, to our knowledge.

We considered corresponding authors rather than first or last authors under the assumption that the corresponding author received the original commentary invitation. Sole authors were assumed to be the corresponding author. Multiauthor articles with no corresponding author were excluded.

### Exposure

The exposure was author gender, treated for the purpose of this analysis as binary (ie, male or female). Gender was inferred from author first name and country of origin using the software service genderize.io^34^ and several supplementary steps (eAppendix 1 in the Supplement). This process returned 1 of 3 results: male, female, or unknown.

### Journal and Article Inclusion Criteria

**Figure 1.**
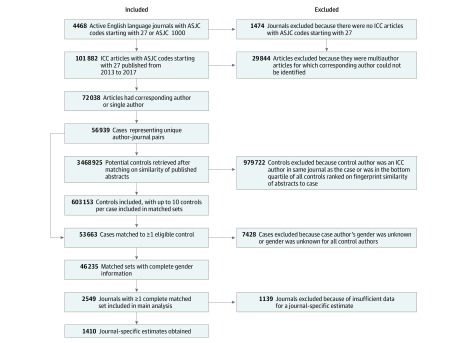
Inclusion and Exclusion Criteria and Sources of Missing Data ASJC indicates All Science Journal Classification; ICC, intraciting commentary.

Inclusion and exclusion criteria are shown in Figure 1. We included journals that publish in English and were listed as active in Scopus at data extraction on December 5, 2018. To ensure our analysis reflected current practice, we included only ICCs published from January 1, 2013, through December 31, 2017. Data analyses were conducted from March 13, 2019, to May 3, 2019.

We restricted our analysis to medical journals (All Science Journal Classification [ASJC] codes starting with 27) and multidisciplinary journals (ASJC code 1000). For medical journals, we included all ICC articles. For multidisciplinary journals, we included only medical ICC articles (article ASJC codes starting with 27); these publications were included to capture medical research published in high-profile general science journals.

Sample size was determined by the number of journals and ICCs in those journals meeting our inclusion criteria (Figure 1).

### Study Design

We used a matched case-control study design to estimate the odds ratio (OR), *γ_j_*, of authoring an ICC in journal *j* for women compared with men. Cases were defined as corresponding or sole authors of 1 or more ICC article published in *j* from January 1, 2013, through December 31, 2017. Potential controls were authors of at least 5 articles (any authorship position) published in any Scopus-indexed journal from January 1, 2013, through December 31, 2017, who were not also cases for journal *j*. Case authors could act as controls for ICC authors published in a different journal but could not act as controls for other case authors within the same journal. Thus, each matched set corresponded to a unique corresponding author-journal pair, so that authors of ICCs in *m* > *1* distinct journals during the study period contributed *m* cases to the data set.

We controlled for key author-level factors, such as field of expertise, seniority, number of publications, and citation record, that may have been associated with gender and ICC authorship. Gender ratios can vary across specific fields of expertise within medical disciplines,^1^ and we hypothesized that some fields would attract more ICCs. We further hypothesized that ICCs were most likely to be authored by senior researchers with strong publication records, factors also associated with gender.

### Control for Author-Level Factors

We matched controls to cases on field of expertise. This was achieved by generating an expertise profile for each author via natural language processing of all abstracts published January 1, 2013, through December 31, 2017, in Scopus-indexed journals (eFigure 1 and eFigure 2 in the Supplement). We compared the expertise profiles of all Scopus-indexed authors with each case author and generated a similarity index for each pair. Full details on our matching method are presented in the eAppendix 1 in the Supplement. The top 50 matches based on this index were potential controls for each case author. To ensure a minimum level of match quality, we pooled potential controls for all cases, ranked on expertise similarity to their respective cases, and excluded the overall lowest quartile. The top 10 remaining controls per case were included in our final data set.

We also controlled for years active (a measure of seniority, defined as years since first publication), h-index (a measure of author citation impact), and number of publications. Percentile ranks of these variables were computed and included in our regression models. Percentile ranks were used to reduce the effect of outliers on our data.

### Statistical Analysis

First, we concatenated the data for all journals, including those with sample sizes too small for journal-specific estimation, and estimated the overall OR using conditional logistic regression. Authors whose gender could not be inferred were excluded from all analyses. We ran 5 conditional logistic regression models including the following independent variables: (1) only gender; (2) gender and percentiles of years active, h-index, and number of publications, allowing for nonlinearity using natural cubic splines with internal knots at 0.25, 0.50, and 0.75; and (3) 3 separate models adding an interaction between gender and the linear term for each continuous variable. All models controlled for field of expertise through matching.

Next, we estimated *γ_j_*, the OR for journal *j*, separately for each journal with sufficient data for an individual estimate. Owing to smaller sample sizes, we controlled for continuous variables using polynomial terms rather than splines (eAppendix 1 in the Supplement).

To investigate the robustness of our overall OR estimate to potential sources of error, we performed 3 sensitivity analyses (eAppendix 1 in the Supplement). First, we used random effects meta-analysis to account for possible between-journal heterogeneity in the OR.^35^ Second, we used multiple imputation to account for missing gender. Third, we increased the stringency of criteria for matching on field expertise, excluded ICCs that may have been replies to other articles, and excluded duplicate records for authors of ICCs in multiple journals.

To examine possible sources of heterogeneity in the magnitude of the association, we performed 2 secondary analyses. First, to investigate whether *γ_j_* was associated with the journal citation impact as measured by 2016 CiteScore (mean number of citations received from 2014 through 2016 for all items published from 2014 through 2016), we included an interaction between gender and CiteScore in our pooled conditional logistic regression models. We allowed for nonlinearity using natural cubic splines with external knots at the maximum and minimum CiteScore in our data set and 2 internal knots at the tertiles of ICC source journal CiteScore. We tested the null hypothesis of no interaction between gender and CiteScore using a likelihood ratio test.

Second, we investigated whether log(*γ_j_*) varied across medical journal topics as defined by ASJC codes. Scopus-indexed journals are assigned 1 or more ASJC code according to the scientific content of a typical volume. We concatenated the data for sets of journals with each ASJC code to obtain a topic-specific estimate. Topics were overlapping because many journals have multiple codes; we were therefore unable to perform statistical tests for differences between topics. All analyses were performed in R statistical software version 3.4.2 (R Project for Statistical Computing). *P* values were 2-sided, and statistical significance was set at .05.

## Results

Deidentified matched data and code are available elsewhere.^36^ We identified 72 038 ICCs that met our inclusion criteria (Figure 1). Gender could be inferred for corresponding authors of 62 567 ICCs (86.9%), 14 140 of whom were female (22.6%). eTable 1 in the Supplement presents characteristics of male, female, and unknown gender authors.

After removing authors with missing gender and other processing steps, our analysis included 2549 journals with a total of 46 235 matched sets (Figure 1). The median (interquartile range [IQR]) number of controls per matched set was 8 (5-9) articles. For 11 047 matched sets with female case authors, the median (IQR) percentage of female controls was 40.0% (22.2%-55.6%). For 35 188 matched sets with male case authors, the median (IQR) percentage of female controls was 25.0% (11.1%-40.0%).

Our data set included 34 047 unique ICC authors, among whom 9072 (26.6%) were female. There were 7721 ICC authors (22.7%) who had published in more than 1 journal. Compared with authors who served only as controls, authors who served only as cases or as both cases and controls had higher median (IQR) years since first publication (control only: 17 [11-27] years; case only: 22 [14-32] years; case and control: 24 [16-33] years), numbers of publications (control only: 60 [30-119] publications; case only: 77 [41-142] publications; case and control: 147 [81-257] publications), and h-indices (case only: 17 [10-29]; control only: 22 [12-36]; case and control: 33 [21-50]) (Table). We had missing data for 1 or more of these variables for 134 controls (0.1%) and no cases.

**Table.** Author-Level Variables by Case-control Status for Unique Authors Included in Analyses

Variable	Median (IQR)			
	Case	Control	Both Case and Control^a^	All
Gender, No. (%)				
Male	10 975 (71.1)	56 829 (63.4)	14 000 (75.2)	81 804 (66.1)
Female	4463 (28.9)	32 793 (36.6)	4609 (24.8)	41 865 (33.9)
Actively publishing, y	22 (14-32)	17 (11-27)	24 (16-33)	19 (12-28)
Publications	77 (41-142)	60 (30-119)	147 (81-257)	71 (35-142)
H-index	22 (12-36)	17 (10-29)	33 (21-50)	19 (11-33)
Total, No.	15 438	89 622	18 609	123 669

Abbreviation: IQR, interquartile range.

^a^ Case authors may also act as controls for other case authors published in a different journal.

### Association of Gender With ICC Authorship

Controlling for field of expertise only, the estimated OR for women compared with men was 0.70 (95% CI, 0.68-0.72; *P* < .001). After adjustment for percentiles of years active, h-index, and number of publications, the OR estimate was 0.78 (95% CI, 0.76-0.80; *P* < .001). Full results of these analyses are presented in eTable 2 and eFigure 3 in the Supplement.

**Figure 2.**
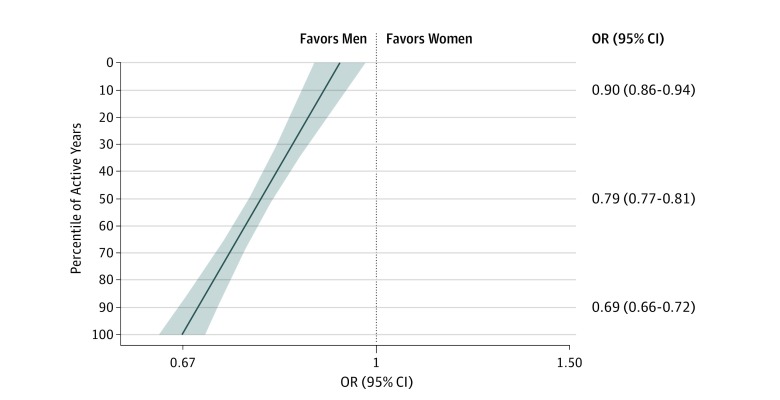
Odds of Authoring an Invited Commentary for Women vs Men by Percentile of Years Active Results are adjusted for field of expertise, h-index percentile, and total number of publications percentile. The 10th percentile indicates approximately 8 years; the 50th percentile, approximately 19 years (the median number); 90th percentile, approximately 38 years; line, odds ratio (OR); shading, 95% CI.

After including an interaction between gender and years active, an increase of 1 decile in years active was associated with a decrease in OR by a factor of 0.97 (95% CI, 0.96-0.98; *P* < .001). Full results of these analyses are presented in eTable 3 and eFigure 4 in the Supplement. The estimated OR among researchers who had been actively publishing for approximately 8 years (ie, 10th percentile) was 0.90 (95% CI, 0.86-0.94; *P* < .001) (Figure 2). At 19 years of actively publishing (50th percentile), the OR was reduced to 0.79 (95% CI, 0.77-0.81; *P* < .001). At 38 years of actively publishing (90th percentile), the OR was 0.69 (95% CI, 0.66-0.72; *P* < .001) (Figure 2).

Results were similar in models including interactions for gender with h-index and gender with number of publications (eTable 4, eTable 5, and eFigures 5-7 in the Supplement). This result is likely associated with the high pairwise correlation between years active, h-index, and number of publications (Spearman correlation coefficient: years active and h-index, 0.72; years active and number of publications, 0.70; h-index and number of publications, 0.91).

There were 1410 journals (55.3%) with sample sizes large enough for journal-specific OR estimates. These analyses are presented in eAppendix 3 in the Supplement, including yearly ICC numbers alongside journal-specific ORs.

### Sensitivity Analyses

Point estimates and 95% CIs from sensitivity analyses were close to those from the main analyses. These analyses are presented in the eAppendix 2, eTables 6-8, eFigure 8, and eFigure 9 in the Supplement.

### Interactions With Journal Topic and CiteScore

**Figure 3.**
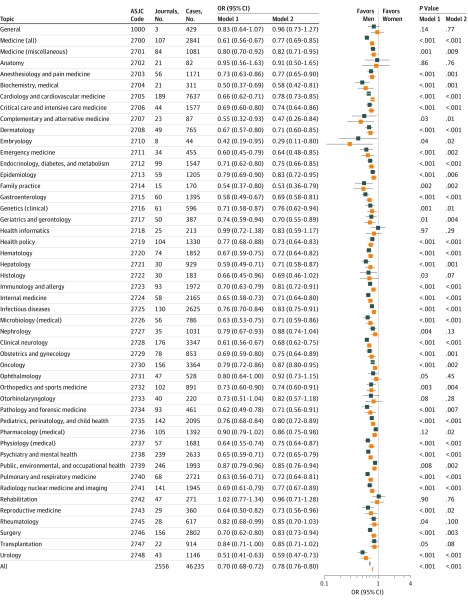
Subgroup Analyses by Journal Topic Journals may have multiple All Science Journal Classification (ASJC) codes; thus, the topics overlap with respect to journals included. Model 1 (orange squares) controls for authors’ fields of expertise through matching. Model 2 (blue squares) includes the adjustments in model 1 and also adjusts for years active percentile, h-index percentile, and total number of publications percentile. Years active indicates years since first publication in Scopus; OR, odds ratio; error bars, 95% CIs.

Subgroup analyses by journal topic yielded statistically significant adjusted ORs favoring male authors for 38 of 48 topics (79%) (Figure 3). The multidisciplinary journals (ASJC code 1000), which had a pooled OR of 0.96 (95% CI, 0.73-1.27), were a notable exception.

**Figure 4.**
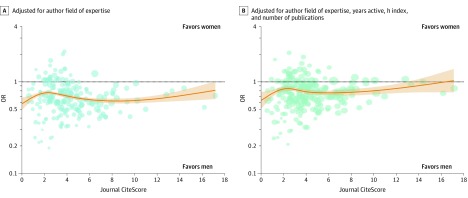
Journal-Specific Odds Ratios (ORs) Plotted Against CiteScore The line and shading represent the OR and 95% CI, respectively, for women vs men of authoring invited commentaries as a function of the CiteScore of the journal in which the commentary was published. Each circle represents the OR estimated for a single journal, and circle diameter is inversely proportional to the SE of the log OR estimate. Only journals with more than 50 matched sets are shown.

The association of OR with journal CiteScore was nonmonotonic (Figure 4) and statistically significant (adjusted for field of expertise: χ^2^_4_, 35.1; *P* < .001; fully adjusted: χ^2^_4_, 35.2; *P* < .001). However, the odds favored men at all CiteScores (Figure 4). One journal with an outlier CiteScore was excluded (*CA: A Cancer Journal for Clinicians*; CiteScore = 89.23).

## Discussion

In this matched case-control study, we estimated the odds ratio of authoring an invited commentary in a medical journal for women compared with men who had similar scientific expertise, seniority, and publication metrics. We observed a statistically significant association in the odds for female researchers, which were approximately 21% lower than for their male peers. This result challenges the common assumption that gender disparities in invited article authorship can be explained by greater publication success, seniority, or self-selection into competitive fields among male scientists. The gender disparity in our study was larger for researchers who had been actively publishing for longer, increasing by appoximately 3% for every 1-decile increase in years active (Figure 2). The odds of invited commentary authorship consistently favored men for journals across the spectrum of citation impact (Figure 4) and for most medical subdisciplines (Figure 3). These results may be generalizable to English-language medical journals, but gender disparities can vary geographically.^16^ We obtained individual estimates for 1410 journals. Deidentified, matched data, and code for analyses are available elsewhere.^36^

Only 23% of invited commentaries in our data set had women corresponding authors, which was consistent with the literature on gender disparities in solicited article authorship.^10,22,25,27,28^ Our study suggested that this gap may be explained by gender-dependent factors unrelated to field of expertise, seniority, or publication impact, characteristics we expected to motivate commentary invitations. One possible explanation is that women declined invitations more frequently. Previous studies have suggested that women academics have lower access to resources,^37^ experience greater role overload,^38^ and take on a higher burden of nonresearch tasks.^39,40^ These factors could reduce their capacity or willingness to accept commentary invitations. Women scientists may also decline invitations more than men owing to lower self-perceived expertise. For future research, journals could keep records of invitations to establish whether there are gender differences in acceptance rates.

Another possible explanation for our results was the presence of gender bias in commentary invitations from publishing gatekeepers. Overt discrimination against women scientists persists in some settings,^41^ and its effect cannot be ruled out. However, gender bias often takes subtler forms. Unconscious gender bias favoring men has been shown to affect evaluations of scientific competency^42,43^ and manuscript quality^44,45,46^ in some contexts. Men editors may have favored men authors owing to same-gender preferences in professional networks.^13,26^ Combined with the overrepresentation of men editors,^13,17,18^ this could have boosted invited authorship by men. Additionally, some journals invite reviewers of research articles to write commentaries. Since women review research articles less frequently,^15^ this mechanism may also have favored men. Many large journals dedicate an editor to commentaries and editorials, while smaller journals often assign this role to editors with less time to devote to the task. Time-pressured editors may rely more on professional contacts, reviewers, or instinctive impressions to select commentary authors, increasing vulnerability to bias. This difference could help explain the slightly smaller gender disparity for large, high-impact journals observed in our study (Figure 4).

In our study, gender disparities in invited commentary authorship increased with author seniority and publication metrics. We offer 2 interpretations for this finding. First, commentary authors may have been more likely to be solicited for future articles, causing gender differences among junior researchers to increase through time. This phenomenon, whereby success begets success, is known as the *Matthew effect*^47^ and has been observed in science funding outcomes.^48^ Second, the benefits of seniority and high-impact publications may have been greater for men than for women. The systematic underrecognition of women scientists’ work, known as the *Matilda effect*,^49^ has also manifested in lower transition rates to principal investigator status for women compared with men who have similar publication records.^50^

### Strengths and Limitations

Our study has several strengths. First, we controlled for field of expertise through matching, an approach that is insensitive to modeling assumptions, and for years active and publication metrics using flexible nonlinear methods. This adjustment allowed us to establish that gender disparities in invited commentary authorship could not be explained by gender differences in these variables. To our knowledge, this is the first study of gender in academic publishing to control for field of expertise using natural language processing. Most previous studies of the association of gender with authorship in medical journals, either invited or contributed, did not control for author-level covariates.^8,10,11,12,13,14,22,28,29^ Some medical specialty–specific studies applied basic controls for a small number of author-level variables.^3,4,5,6,7^ Second, using ICC articles as our outcome allowed us to study a particular type of solicited article for a large number of medical journals and with a large sample size. Third, to our knowledge, our study is the first to provide journal-specific estimates of gender disparities for a large number of medical journals using consistent methods alongside all-journal averages. Journal-specific estimates are important for editorial boards seeking to address gender bias, and consistent methods are needed for between-journal comparisons. Overall estimates are required to obtain a broad view of the field.

Our study also had some limitations. First, we used genderize.io^34^ to infer the corresponding author’s gender from first name and country of origin. Genderize.io performs well compared with similar software,^51^ although all such software is subject to error. Gender could not be inferred for 21% of unique authors. However, multiple imputation analyses did not change our conclusions. We analyzed gender for corresponding authors rather than other authorship positions, assuming that the corresponding author received the commentary invitation. This assumption may not hold for all articles.

Second, we used published abstracts to match fields of expertise. Publications are not the only measure of expertise, and this matching process was imperfect. Anecdotally, controls were often repeat coauthors of cases. Due to gender homophily in coauthorship,^52^ this pattern would likely artificially lower gender diversity within matched sets, attenuating our OR estimates toward the null. We also controlled for publication metrics and years since first publication as a measure of seniority. Publications and citations may have accrued in an environment in which women were systematically disadvantaged.^53^ Women scientists may therefore have been more qualified in general to provide commentary in their respective field than men who were similar on these measures, biasing the estimated OR in favor of women.

Third, our outcome included only ICCs; we did not expect these to be systematically different from other invited commentaries. However, ICCs may have been (1) written by in-house writers, including editors; (2) volunteered by reviewers of research articles; or (3) replies to other articles. While authors of these article types were preselected by the journal, we could not know whether they were invited. We could not ascertain the prevalence of types 1 and 2 with the available data. Removing possible reply articles (type 3) had minimal effect on our results. We excluded articles with missing corresponding author, journal volume, or journal issue information. This administrative missingness was unlikely to bias our overall results. However, it may have rendered some journal-specific estimates unreliable, as missingness varied by year for some journals, likely due to changes at the journal or in Scopus recording methods.

Fourth, our statistical analysis did not account for correlation due to repeated observations from the same author or journal. However, sensitivity analyses suggested this affected our results minimally.

## Conclusions

This study suggests that women had lower odds of authoring invited commentaries than men with similar expertise and scientific standing. Invited commentaries confer career advantages on the author by providing exposure and fostering professional connections with editors. These benefits may accumulate if invited article authors are more likely to be solicited for future pieces. Extending article invitations to researchers primarily based on seniority, perceived prestige, and professional connections may contribute to male scientists’ entrenched advantage and compound gender inequity. Our results suggested that female talent was underused. As such, increasing gender diversity in solicited article authorship may increase the quality of these articles.

Our study indicated one possible way forward: journals could use natural language processing of bibliographic databases to identify experts on invited paper topics. This approach could alert editors to highly qualified author candidates they otherwise might not have considered. Our study provides proof-of-concept for this proposal because we used natural language processing to identify thousands of women with relevant expertise on previous commentary topics and showed that they were underrepresented as commentary authors compared with their male peers. Future work should focus on developing fair, data-driven approaches to expert-finding that avoid propagating biases in bibliographic data.^54^ If successfully implemented, this strategy could diversify professional networks, strengthen the author talent pool, and provide opportunities to a wider group of scientists.

## Notes

Supplement. **eAppendix 1. **Supplementary Methods **eAppendix 2. **Supplementary Results **eFigure 1.** Example of Concepts (Noun Phrases) Identified in an Abstract **eFigure 2.** Articles With Ranked Concepts (Noun Phrases) and Author Profile for Dr Francesca Dominici **eTable 1.** Gender by Author-Level Variables for Unique Authors, Including Authors With Unknown Gender **eTable 2.** Results From Pooled Conditional Logistic Regression Models **eFigure 3.** Odds Ratio for Invited Commentary Authorship as a Function of Years Active, H-Index, and Number of Publications **eTable 3.** Results From Conditional Logistic Regression Model Including Interaction Between Gender and Years Active **eFigure 4.** Results From Conditional Logistic Regression Model Including Interaction Between Gender and Years Active **eTable 4.** Results From Conditional Logistic Regression Model Including Interaction Between Gender and H-Index **eFigure 5.** Results From Conditional Logistic Regression Model Including Interaction Between Gender and H-Index **eTable 5.** Results From Conditional Logistic Regression Model Including Interaction Term Between Gender and Number of Publications **eFigure 6.** Results From Conditional Logistic Regression Model Including Interaction Term Between Gender and Number of Publications **eFigure 7.** Association of Gender With Authoring an Invited Commentary **eAppendix 3.** Estimated Journal-Specific Odds Ratios vs Journal CiteScore **eTable 6. **Association of Gender With Invited Commentary Authorship Esimated via 2-Stage Random Effects Meta-analysis **eFigure 8.** 2-Stage Meta-analysis Results by Journal Topic **eFigure 9.** Meta-regression of Journal-Specific Effect Sizes Against CiteScore **eTable 7.** Association of Gender With Invited Commentary Authorship Accounting for Missing Gender Data via Multiple Imputation **eTable 8.** Association of Gender on Invited Commentary Authorship After Excluding Duplicates for Case Authors Who Appear in Multiple Journals, Excluding Articles That May Be Replies to Other Articles, and Keeping Only Top 2 Controls Per Case **eReferences**
