# Supplementary material for: Gender Disparities in Invited Commentary Authorship in 2459 Medical Journals
Source: JAMA Netw Open. 2019 Oct 23;2(10):e1913682. doi: 10.1001/jamanetworkopen.2019.13682 (PMC6820037; doi:10.1001/jamanetworkopen.2019.13682)
Supplement: Supplement. — eAppendix 1. Supplementary Methods eAppendix 2. Supplementary Results eFigure 1. Example of Concepts (Noun Phrases) Identified in an Abstract eFigure 2. Articles With Ranked Concepts (Noun Phrases) and Author Profile for Dr Francesca Dominici eTable 1. Gender by Author-Level Variables for Unique Authors, Including Authors With Unknown Gender eTable 2. Results From Pooled Conditional Logistic Regression Models eFigure 3. Odds Ratio for Invited Commentary Authorship as a Function of Years Active, H-Index, and Number of Publications eTable 3. Results From Conditional Logistic Regression Model Including Interaction Between Gender and Years Active eFigure 4. Results From Conditional Logistic Regression Model Including Interaction Between Gender and Years Active eTable 4. Results From Conditional Logistic Regression Model Including Interaction Between Gender and H-Index eFigure 5. Results From Conditional Logistic Regression Model Including Interaction Between Gender and H-Index eTable 5. Results From Conditional Logistic Regression Model Including Interaction Term Between Gender and Number of Publications eFigure 6. Results From Conditional Logistic Regression Model Including Interaction Term Between Gender and Number of Publications eFigure 7. Association of Gender With Authoring an Invited Commentary eAppendix 3. Estimated Journal-Specific Odds Ratios vs Journal CiteScore eTable 6. Association of Gender With Invited Commentary Authorship Esimated via 2-Stage Random Effects Meta-analysis eFigure 8. 2-Stage Meta-analysis Results by Journal Topic eFigure 9. Meta-regression of Journal-Specific Effect Sizes Against CiteScore eTable 7. Association of Gender With Invited Commentary Authorship Accounting for Missing Gender Data via Multiple Imputation eTable 8. Association of Gender on Invited Commentary Authorship After Excluding Duplicates for Case Authors Who Appear in Multiple Journals, Excluding Articles That May Be Replies to Other Articles, and Keeping Only Top 2 Contro [file jamanetwopen-2-e1913682-s001.pdf]

Supplementary Online Content

Thomas EG, Jayabalasingham B, Collins T, Geertzen J, Bui C, Dominici F. Gender disparities in invited commentary authorship in 2459 medical journals. *JAMA Netw Open*. 2019;2(10):e1913682. doi:10.1001/jamanetworkopen.2019.13682

eAppendix 1. Supplementary Methods
eAppendix 2. Supplementary Results
eFigure 1. Example of Concepts (Noun Phrases) Identified in an Abstract
eFigure 2. Articles With Ranked Concepts (Noun Phrases) and Author Profile for Dr Francesca Dominici
eTable 1. Gender by Author-Level Variables for Unique Authors, Including Authors With Unknown Gender
eTable 2. Results From Pooled Conditional Logistic Regression Models
eFigure 3. Odds Ratio for Invited Commentary Authorship as a Function of Years Active, H-Index, and Number of Publications
eTable 3. Results From Conditional Logistic Regression Model Including Interaction Between Gender and Years Active
eFigure 4. Results From Conditional Logistic Regression Model Including Interaction Between Gender and Years Active
eTable 4. Results From Conditional Logistic Regression Model Including Interaction Between Gender and H-Index
eFigure 5. Results From Conditional Logistic Regression Model Including Interaction Between Gender and H-Index
eTable 5. Results From Conditional Logistic Regression Model Including Interaction Term Between Gender and Number of Publications
eFigure 6. Results From Conditional Logistic Regression Model Including Interaction Term Between Gender and Number of Publications
eFigure 7. Association of Gender With Authoring an Invited Commentary
eAppendix 3. Estimated Journal-Specific Odds Ratios vs Journal CiteScore
eTable 6. Association of Gender With Invited Commentary Authorship Esimated via 2-Stage Random Effects Meta-analysis
eFigure 8. 2-Stage Meta-analysis Results by Journal Topic
eFigure 9. Meta-regression of Journal-Specific Effect Sizes Against CiteScore
eTable 7. Association of Gender With Invited Commentary Authorship Accounting for Missing Gender Data via Multiple Imputation
eTable 8. Association of Gender on Invited Commentary Authorship After Excluding Duplicates for Case Authors Who Appear in Multiple Journals, Excluding Articles That May Be Replies to Other Articles, and Keeping Only Top 2 Controls Per Case
eReferences.

This supplementary material has been provided by the authors to give readers additional information about their work.

© 2019 Thomas EG et al. *JAMA Network Open.*

eAppendix 1. Supplementary Methods

Gender inference details

In our study, we used a four-step process to infer author gender. In Step 1, we inputted author first name and country of origin to the software service genderize.io.[1] If Step 1 failed, we moved to Step 2: using genderize.io inputting first name only. In Steps 1 and 2, we required the following criteria in order to assign a gender: (a) the name must appear in the genderize.io dictionary at least five times; (b) the probability of that name being either male or female must be 85% or more according to the statistics provided by genderize.io.

If Steps 1 and 2 both failed to return a gender, we moved to Step 3, which matched author first and last name to a dictionary of names and countries from the journal Nature. Finally, if Steps 1, 2, and 3 all failed, in Step 4 we matched first names to a dictionary of Japanese first names and their genders. We note that in tests using a set of names with known genders, Steps 3 and 4 did not significantly improve accuracy of gender inference.

Matching algorithm details

Technical details

We identified potential controls based on published article abstracts available in Scopus, the largest abstract and citation database of peer-reviewed literature. The matching algorithm accepts as input the set of all available Scopus abstracts (no restrictions on journal) and produces as output a measure of the similarity of the abstracts of all authors in Scopus to the abstracts of each case author. The degree of similarity determined by the algorithm depends both on the semantic concepts identified in an author's abstracts, as well as the frequency with which the author produces abstracts that refer to that concept. Typical controls thus either publish prolifically on at least one topic that the case author works on (a strong match on some concepts) or publish to some extent on *all* concepts that the case author works on (a moderate match on all concepts), or a balance of the two.

Each Scopus publication was characterized by a set of noun phrases extracted from title and abstract, where the term “noun phrase” refers to a group of words that behaves like a noun and often has a noun as nucleus (see eFigure 1(a) and (b) for examples). The term frequency (TF) of a noun phrase was multiplied by its inverse document frequency (IDF) to obtain the TF-IDF, a well-validated measure of how important the phrase is to the publication in the collection of all publications in the time range of interest. Within each document, noun-phrases were then ranked according to TF-IDF value (see eFigure 1(c) and eFigure 2(a) for examples). Note that the noun-phrases were identified from *all* abstracts available in Scopus, not just abstracts from the journal where the case ICC was published.

Next, author expertise profiles were generated by computing the rank of each phrase, averaged across all of the author's publications, as a measure of the importance of that phrase for the author (see eFigure 2(b) for an example). Only authors that had at least five publications in the time range of interest (2013 through 2017) were included to ensure a sufficiently rich semantic representation. Rare noun phrases that occurred in only a few documents across the whole database were not included in the profiles. We then compared the profile of each case author to the profiles of all other authors in the database using the BM25 ranking function,[2,3] which has shown strong performance in information retrieval ranking tasks. The top 50 most similar authors for each case were taken as potential matched controls.

Validity of the matching algorithm

We used TF-IDF and BM25 to rank potential controls for each case author based on published abstracts. This system has previously been used in expert search tasks similar to our application,[4] has been well-tested experimentally in a wide range of other document information retrieval tasks,[5,6] and typically rivals or outperforms competing algorithms.[7] A previous study using TF-IDF/BM25 and the same natural language processing methods used here demonstrated good performance in matching unsubmitted manuscripts to potential journals.[8]

© 2019 Thomas EG et al. *JAMA Network Open.*

Comparison with other text matching methods

From other tools that implement text similarity, the approach adopted in this study is most similar to the system Jane, which uses text similarity to suggest journals and experts.[9] The most important algorithmic differences concern the ranking function, the modelling of the text, and the matching at researcher level. The Jane system uses plain TF-IDF values whereas the current approach uses a more powerful ranking function (BM25). Importantly, Jane does only model isolated words (e.g. radon, exposure) whereas the current approach captures meaningful compound words and phrases (e.g. radon_exposure) that better describe the content. Finally, the current approach aggregates to researcher profiles first, carefully balancing the contribution of individual articles. An in-depth comparison is beyond the scope of this study, but each of the differences with respect to Jane is known to contribute towards higher accuracy in semantic similarity matching.[2,7]

Estimation of journal-specific odds ratios

Estimation of journal-specific odds ratios involved fitting conditional logistic regression models separately to data for each journal. Three models were fit for each journal: Model 1: effect of gender adjusted only for field of expertise through matching; Model 2: further adjusted for percentiles of years active, h-index, and number of publications as covariates in the regression model, and; Model 3: including an interaction term between years active percentile and gender.

For Models 2 and 3, most journals did not have sufficient data to permit use of spline terms, as for our pooled model using data from all journals (see Statistical Analyses section of main manuscript). Based on the functional forms for the effects of years active, h-index and number of publications estimated in our pooled model (see eFigures 3 and 4), we included the following terms in Model 2: a linear term for years active percentile, and linear and quadratic terms for each of h-index percentile and number of publications percentile. In Model 3, an interaction term was included between gender and the linear effect of years active percentile.

Due to small sample sizes, we were able to fit Model 1 for 1,410 journals, Model 2 for 1,196 journals, and Model 3 for 1,087 journals. Journal-specific results for Models 1 and 2 can be found in the eAppendix. Results for Model 3 were used for random effects meta-analysis, discussed in the next section.

Sensitivity analyses

Sensitivity analysis 1: two-stage random effects meta-analysis

In our main analysis, we concatenated the datasets for all journals and estimated the overall OR using conditional logistic regression. This approach to combining data from multiple sources (journals, in our study) is known as *one-stage meta-analysis* in the context of meta-analyses where individual-level data are available.[10] In a sensitivity analysis, we compared results from the one-stage meta-analysis to a two-stage meta-analysis. The two-stage approach involved combining estimates of $\log \gamma_j$, the journal-specific log odds ratios, and their estimated variances using random effects meta-analysis. Two-stage meta-analysis accounts for between-journal heterogeneity in exposure and covariate effect sizes, while one-stage meta-analysis is biased in the presence of such heterogeneity.[11] However, the two-stage approach necessarily excluded data from journals with sample sizes too small to permit estimation of $\log \gamma_j$. Estimates of $\log \gamma_j$ were obtained as described in the previous section.

We also repeated our secondary analyses using two-stage meta-analysis. To investigate the effect of journal topic on the odds ratio for gender, we pooled journal-specific estimates using random effects meta-analysis for all journals having particular All Science Journal Classification (ASJC) codes. To investigate the effect of journal citation impact on the odds ratio for gender, we conducted a meta-regression of journal-specific log odds ratios on journal Cite Score.

Sensitivity analysis 2: multiple imputation for missing gender data

Overall, 21.0% (35,230 of 167,705) of unique authors in our dataset could not be assigned a gender. This missingness was related to case status, Asian country of origin, years active, number of publications and h-index (eTable 1).

© 2019 Thomas EG et al. *JAMA Network Open.*

Having unknown gender also may be related to the true unknown gender. Genderize.io is known to return "unknown gender" more often for Asian names,[12] and researchers with Asian names may have a different gender ratio than other researchers.[13,14] If having an Asian name is also related to the chance of authoring an invited commentary, this missingness could bias our results. We defined Asian country of origin as having at least one publication in the author's first year of data in Scopus where the affiliation address could be determined and was in an Asian country. In our dataset, 30,823 (18.4%) unique authors were determined to have Asian country of origin (eTable 1). Gender could not be inferred for 15,743 (51.1%) Asian researchers, compared to 19,054 (14.0%) non-Asian researchers.

We hypothesized that the gender data are approximately missing at random (MAR). Specifically, we claim that missingness in the gender variable is likely to be independent of gender after accounting for author-level characteristics including having an Asian name, case status, years active, number of publications, and h-index. Multiple imputation is therefore an appropriate method to account for missing gender in our data.

We built a mixed effects logistic regression imputation model for gender that included the following variables. Asian country of origin was included as a binary variable. Non-linear effects for percentiles of years active, number of publications and h-index were included using natural cubic splines with internal knots at 0.25, 0.5, and 0.75. For consistency with our outcome model, we also included interactions between case status and the linear terms for each of years active, number of publications, and h-index. Finally, we included a random effect for matched set to account for the association between field of scientific expertise (the matching variable) and gender.

After running the above model, we generated predicted probabilities of being female for all authors with missing gender information. We then generated ten datasets with missing gender imputed randomly based on these probabilities. We ran our conditional logistic regression outcome models using each of these datasets and pooled the regression coefficients using Rubin's rules for multiple imputation.

Sensitivity analysis 3: de-duplication for case authors present in multiple journals, excluding reply articles, and increased stringency of matching criteria

In a third sensitivity analysis, we repeated our main analyses using a dataset based on more conservative assumptions. This allowed us to examine the potential impact of three issues: (1) correlation due to multiple cases representing the same author; (2) the presence of articles that may not have been invited, and; (3) variation in the match quality of controls.

First, in our dataset, one author acts as a case m times if they authored an invited commentary in m distinct journals over the study period. This leads to "duplicate" records for those authors with $m > 1$. These authors may have an inflated impact on our results, especially when m is large. 25.1% of male cases authored ICCs in multiple journals (range of number journals per author: 1 to 22), compared to 16.0% of female cases (range: 1 to 10). To account for this, we removed duplicates so that each author appeared at most once as a case.

Second, our outcome definition, intra-citing commentaries (ICCs), may include some article types that are arguably not invited. Some of these article types could not be identified with the available data (see Limitations section of the manuscript). However, one such article type — replies to other articles — is typically indicated by its title. In many medical journals, authors are given the opportunity to respond to commentaries or letters concerning an article they have authored, and this response may be published alongside the commentary/letter. These replies typically include phrases like "response to", "a reply", or "authors' response", etcetera, in their titles, and cite the article they are responding to, such that they meet our definition of an ICC. We searched for articles in our dataset with titles containing at least one of the words "reply", "replies", "response", "responses", "respond", or "responds". This definition was chosen to be inclusive in order to capture the maximum number of reply articles; however, articles with these words in their titles are not necessarily replies. There were 9,354 such articles in our dataset (9.2% of eligible articles). These articles were excluded.

Third, in our main analysis, we included up to ten controls per case. The quality of the match between each control and the corresponding case depends on the similarity index generated when comparing Scopus abstracts. This similarity index does not have interpretable units; hence, the choice of cut-off for this index is somewhat arbitrary.

© 2019 Thomas EG et al. *JAMA Network Open.*

We investigated the impact of increasing the stringency of matching criteria by keeping only the top two controls per case based on similarity index.

© 2019 Thomas EG et al. *JAMA Network Open.*

eAppendix 2. Supplementary Results

Sensitivity analyses

Sensitivity analysis 1: two-stage random effects meta-analysis

After excluding 1,139 journals that had insufficient data to obtain a journal-specific estimate, the random effects meta-analysis included data from 1,410 journals with a total of 43,572 matched sets. Adjusted results, shown in eTable 6, were very similar to those of our one-stage meta-analysis, shown in eTables 2 and 3.

eFigure 8 shows that topic-specific ORs were broadly similar to those from our sub-group analysis using one-stage meta-analysis, shown in Figure 3 of the main text. eFigure 9 shows that the association between journal-specific ORs and journal Cite Score estimated using meta-regression was very similar to the analogous one-stage result shown in Figure 4 of the main text.

Sensitivity analysis 2: multiple imputation for missing gender data

Results from multiple imputation analyses are shown in eTable 7. Accounting for missing gender data using multiple imputation slightly increased the magnitude of our point estimates. The odds ratio adjusted for field of expertise, years active, h-index, and number of publications was 0.76 (95%CI: 0.74 to 0.78) after accounting for missing data, compared to 0.78 (95%CI: 0.76 to 0.80) in a complete case analysis.

Sensitivity analysis 3: de-duplication for case authors present in multiple journals, excluding reply articles, and increased stringency of matching criteria

After excluding duplicate records for the same author, excluding possible reply articles, and keeping only the top two most closely matched controls per case, 31,821 matched sets were included in this sensitivity analysis. eTable 8 shows that results were very similar to our original analysis.

© 2019 Thomas EG et al. *JAMA Network Open.*

eFigure 1. Example of Concepts (Noun Phrases) Identified in an Abstract

(a) Article abstract[15]

American journal of epidemiology
Volume 188, Issue 5, 1 May 2019, Pages 866-872

The Future of Climate Epidemiology: Opportunities for Advancing Health Research in the Context of Climate Change (Article)

Anderson, G.B.[a], Barnes, E.A.[b], Bell, M.L.[c], Dominici, F.[d]

Abstract

In the coming decades, climate change is expected to dramatically affect communities worldwide, altering the patterns of many ambient exposures and disasters, including extreme temperatures, heat waves, wildfires, droughts, and floods. These exposures, in turn, can affect risks for a variety of human diseases and health outcomes. Climate epidemiology plays an important role in informing policy related to climate change and its threats to public health. Climate epidemiology leverages deep, integrated collaborations between epidemiologists and climate scientists to understand the current and potential future impacts of climate-related exposures on human health. A variety of recent and ongoing developments in climate science are creating new avenues for epidemiologic contributions. Here, we discuss the contributions of climate epidemiology and describe some key current research directions, including research to better characterize uncertainty in climate health projections. We end by outlining 3 developing areas of climate science that are creating opportunities for high-impact epidemiologic advances in the near future: 1) climate attribution studies, 2) subseasonal to seasonal forecasts, and 3) decadal predictions. © The Author(s) 2019. Published by Oxford University Press on behalf of the Johns Hopkins Bloomberg School of Public Health. All rights reserved. For permissions, please e-mail: journals.permissions@oup.com.

(b) Noun phrases in abstract

climate_epidemiology
opportunities
health_research
climate_change
decades
communities
patterns
extreme_temperatures
heat_waves
draughts
floods
exposures
human_diseases
health_outcomes
climate_epidemiology
…

(c) Noun phrases ranked by TF-IDF

1- climate_epidemiology
2- climate_change
3- opportunity
4- climate_science
5- future
6- variety
7- contribution
8- exposure
9- research_direction
10- human_health
…

Caption: Figure (a) shows an example of an abstract by Dr. Francesca Dominici.[15] Figure (b) shows concepts or "noun phrases" identified in the abstract. Figure (c) shows the top ten noun phrases in the abstract ranked by importance to the abstract, quantified by TF-IDF value. See eMethods (matching algorithm details) for further details. Ellipses (…) indicate that the list continues.

© 2019 Thomas EG et al. *JAMA Network Open.*

eFigure 2. Articles With Ranked Concepts (Noun Phrases) and Author Profile for Dr Francesca Dominici

(a) Articles with ranked noun phrases

**Article electronic identifier (EID)**	**Ranked noun phrases**
2-s2.0-85070336543	dementia, pm2, hospitalization, urbanization, ci, hazard_ratio, level, area, fee_for_service_medicare_record, long_term_effect, …
2-s2.0-85066985365	radon_exposure, follow_up, mortality, mortality_risk, condition, association, cohort_study, individual, medicare_beneficiary, …
2-s2.0-85069706966	particle, greater_boston_area, population_cohort, activity, stroke, exposure_metric, inflammation, pm2, …
2-s2.0-85067554579	ard, pm2, ozone, ci, hospital_admission_rate, increase, part, adult, hospital_admission, long_term_exposures, …
2-s2.0-85068933929	people, heat_wave, system, education, heat_wave_alert, korea, deaths, mortality, ci, city, …
2-s2.0-85067525882	amyloidosis, incidence, prevalence_rate, prevalence, heart_failure, principal, ninth_revision_code, diseases, international_classification, …
2-s2.0-85066456565	uncertainty_estimation, causal_inference, scale, challenge, mortality, exposure_timescale, exposure_surrogate, exposure_simulation_output, …
2-s2.0-85064522915	climate_epidemiology, climate_change, opportunity, climate_science, future, variety, contribution, exposure, research_direction, human_health, …
2-s2.0-85064107396	air_pollution_policy, process, evidence, gps, exposure_model, exposure_timescale, exposure_surrogate, exposure_simulation_output, …
2-s2.0-85062817274	r_package, pm2, train, land_use_datum, health_researcher, scalability, satellite, air_pollution_monitor, conjunction, h2o, …
…	…

(b) Expertise profile for Dr. Dominci

1- mortality; 2- pm; 3- air_pollution; 4- exposure; 5- hospitalization; 6- risk; 7- association; 8- city; 9- evidence; 10- model; 11- effect; 12- study; 13- author; 14- approach; 15- ci; 16- county; 17- ozone; …

Caption: Figure (a) shows ten articles authored by Dr. Francesca Dominici (any authorship position) with their Scopus electronic identifier (EID). The right column shows concepts or “noun phrases” identified in the articles’ abstracts, ranked by importance to that abstract (quantified by TF-IDF value). Figure (b) shows noun phrases ranked by their average TF-IDF rank across all abstracts authored by Dr. Dominici. These ranked noun-phrases form Dr. Dominici’s expertise profile. See eMethods (matching algorithm details) for further details. Ellipses (…) indicate that the list continues; author profiles typically included hundreds of noun phrases.

© 2019 Thomas EG et al. *JAMA Network Open.*

eTable 1. Gender by Author-Level Variables for Unique Authors, Including Authors With Unknown Gender

**Variable**	**Statistic**	**Male**	**Female**	**Unknown gender**	**All**
Case status	Case (column %)	10,454 (12.0%)	4,265 (9.5%)	2,738 (7.8%)	17,457 (10.4%)
	Control (column %)	62,359 (71.3%)	35,908 (79.8%)	29,455 (83.6%)	127,722 (76.2%)
	Both case and control[a] (column %)	14,641 (16.7%)	4,848 (10.8%)	3,037 (8.6%)	22,526 (13.4%)
Country of origin	Number Asian (column %)	11,527 (13.2%)	3,553 (7.9%)	15,743 (44.7%)	30,823 (18.4%)
	Number not Asian (column %)	75,822 (86.7%)	41,414 (92.0%)	19,054 (54.1%)	136,290 (81.3%)
	Number unknown (column %)	105 (0.1%)	54 (0.1%)	433 (1.2%)	592 (0.4%)
Years active	Median (IQR)[b]	21 (13 to 30)	16 (10 to 24)	15 (10 to 23)	15 (11 to 23)
Number of publications	Median (IQR)[b]	84 (41 to 165)	49 (26 to 93)	58 (29 to 116)	66 (33 to 134)
H-index	Median (IQR)[b]	21 (12 to 36)	15 (9 to 26)	15 (9 to 25)	18 (10 to 32)
**Total**	Number (row %)	87,454	45,021	35,230	167,705

[a]Case authors can also act as controls for other case authors published in a different journal
[b]IQR = interquartile range

Caption: Gender was unknown if we could not infer it from author first name and country of origin as described in the eMethods (gender inference details). Cases are authors who published at least one intra-citing commentary (ICC) article in an eligible journal during the study period (2013 through 2017). Controls were matched to cases based on field of expertise as determined using natural language processing of abstracts. Case authors also can act as controls for other case authors published in a different journal, but they cannot act as controls for case authors published in the same journal. Asian country of origin was defined as having at least one publication in the author's first year of data in Scopus where the affiliation address could be determined and was in an Asian country. Years active was defined as years since first publication in Scopus.

© 2019 Thomas EG et al. *JAMA Network Open.*

eTable 2. Results From Pooled Conditional Logistic Regression Models

Model	Variable	Comparison	Odds Ratio	95%CI
Adjusted for field of expertise only				
	Gender	Female vs. male	0.70	(0.68, 0.72)
Fully adjusted [a]				
	Gender	Female vs. male	0.78	(0.76, 0.80)
	Years active percentile		See eFigure 3[a]	
	H-index percentile		See eFigure 3[a]	
	Number of publications percentile		See eFigure 3[a]	

[a] Years since first publication, h-index, and number of publications were included in models as percentiles and were adjusted for using natural cubic splines to allow for non-linear effects. The odds ratio as a function of these variables is displayed in eFigure 3, since coefficients for spline terms are not interpretable. Numeric results are available from the authors upon request, or by accessing full results at github.com/emgthomas/gender_and_invited_commentaries.

© 2019 Thomas EG et al. *JAMA Network Open.*

eFigure 3. Odds Ratio for Invited Commentary Authorship as a Function of Years Active, H-Index, and Number of Publications

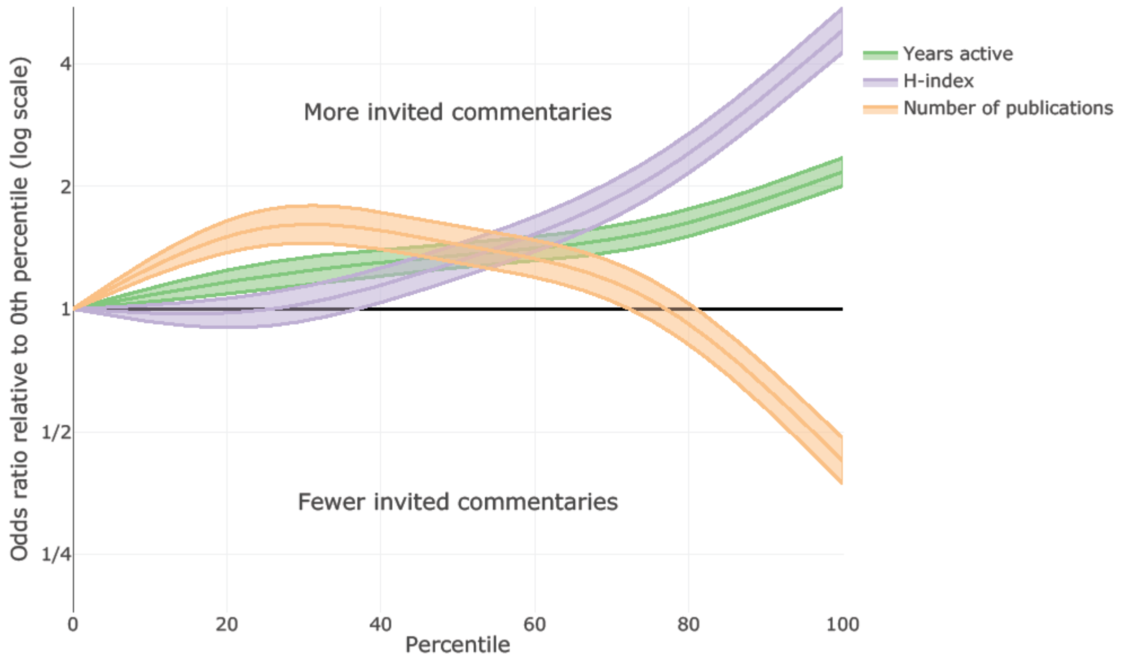

Caption: The figure shows the odds ratio of invited commentary authorship as a function of percentiles of years active, h-index, and number of publications. Results are from a model adjusting for these three variables, as well as adjusting for field of scientific expertise through matching.

© 2019 Thomas EG et al. *JAMA Network Open.*

eTable 3. Results From Conditional Logistic Regression Model Including Interaction Between Gender and Years Active

Variable	Comparison	Odds Ratio	95%CI
Gender	Female vs. male, for authors at lowest percentile of years active	0.93	(0.88, 0.98)
Gender x years active percentile [a]	Female vs. male/10-point increase	0.98	(0.96, 0.98)
Years active percentile [b]		See eFigure 4	
H-index percentile [b]		See eFigure 4	
Number of publications percentile [b]		See eFigure 4	

[a] The interaction between gender and years active is visualized in eFigures 4(a) and 7(a).
[b] Years since first publication, h-index, and number of publications were included in models as percentiles and were adjusted for using natural cubic splines to allow for non-linear effects. The odds ratio as a function of these variables is displayed in eFigure 4, since coefficients for spline terms are not interpretable. Numeric results are available from the authors upon request, or by accessing full results at github.com/emgthomas/gender_and_invited_commentaries.

© 2019 Thomas EG et al. *JAMA Network Open.*

eFigure 4. Results From Conditional Logistic Regression Model Including Interaction Between Gender and Years Active

(a) Odds ratio as a function of years active by gender

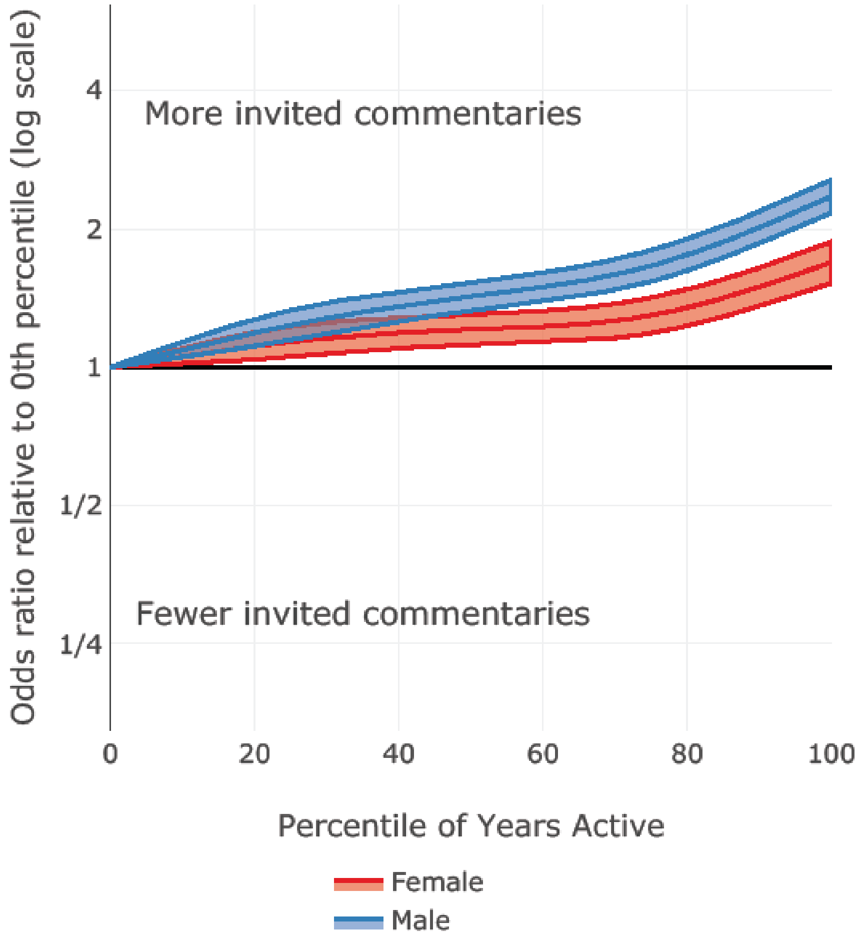

© 2019 Thomas EG et al. *JAMA Network Open.*

(b) Odds ratio as a function of h-index and number of publications

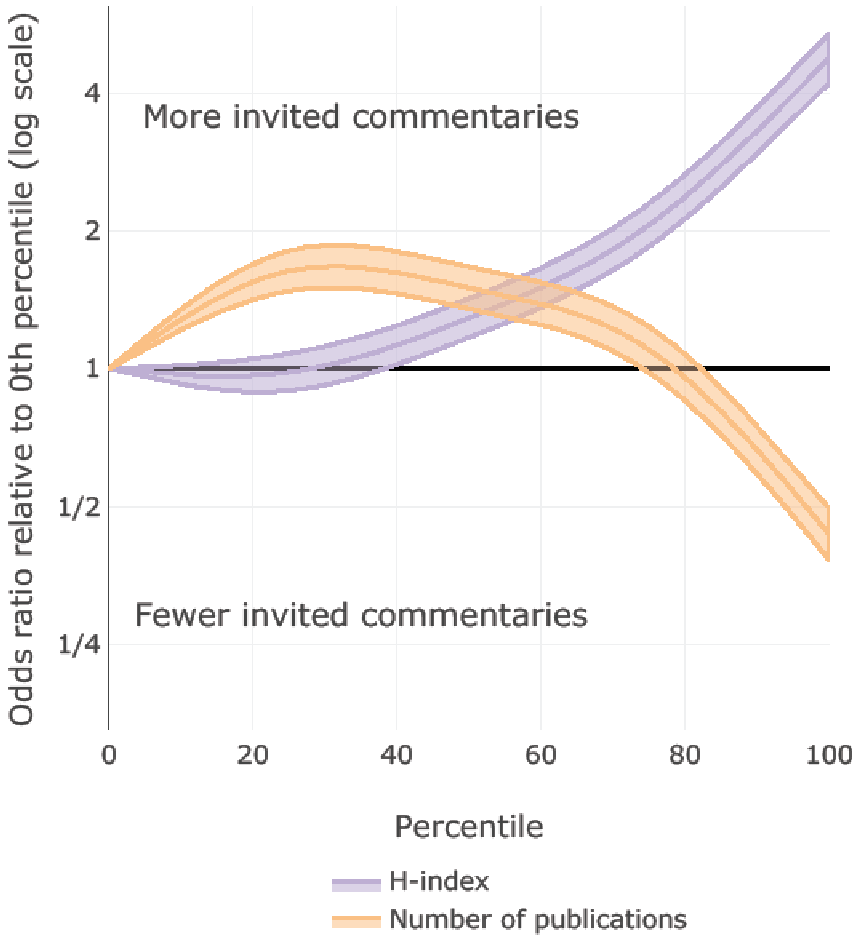

Caption: The figure shows the odds ratio for invited commentary authorship as a function of percentiles of years active, h-index, and number of publications. Results are from a model adjusting for percentiles years active, h-index, and number of publications and allowing for an interaction term between the linear effect of years active and gender. Odds ratios are adjusted for field of expertise through matching.

© 2019 Thomas EG et al. *JAMA Network Open.*

eTable 4. Results From Conditional Logistic Regression Model Including Interaction Between Gender and H-Index

**Variable**	**Comparison**	**Odds Ratio**	**95%CI**
Gender	Female vs. male, for authors at lowest percentile of h-index	0.97	(0.92, 1.03)
Gender x h-index percentile [a]	Female vs. male/10-point increase	0.96	(0.95, 0.97)
Years active percentile [b]		See eFigure 5	
H-index percentile [b]		See eFigure 5	
Number of publications percentile [b]		See eFigure 5	

[a] The interaction between gender and number of publications is visualized in eFigures 5(a) and 7(b).
[b] Years since first publication, h-index, and number of publications were included in models as percentiles and were adjusted for using natural cubic splines to allow for non-linear effects. The odds ratio as a function of these variables is displayed in eFigure 5, since coefficients for spline terms are not interpretable. Numeric results are available from the authors upon request, or by accessing full results at github.com/emgthomas/gender_and_invited_commentaries.

© 2019 Thomas EG et al. *JAMA Network Open.*

eFigure 5. Results From Conditional Logistic Regression Model Including Interaction Between Gender and H-Index

(a) Odds ratio as a function of h-index by gender

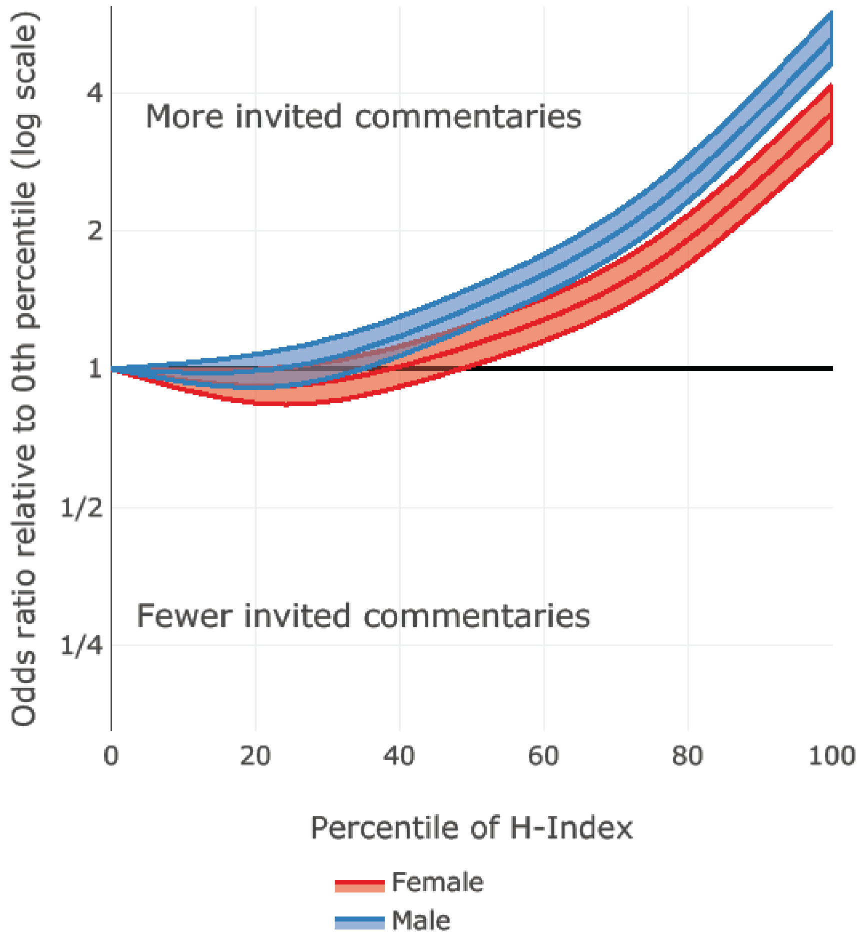

© 2019 Thomas EG et al. *JAMA Network Open.*

(b) Odds ratio as a function of years active and number of publications

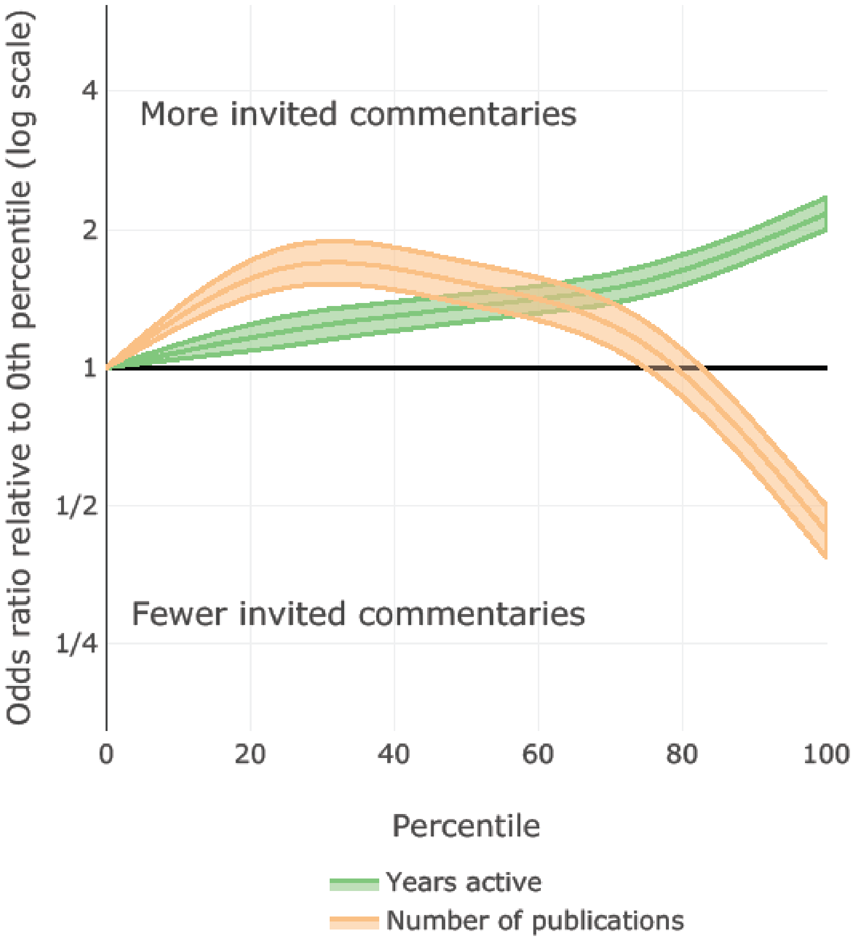

Caption: The figure shows odds ratio for invited commentary authorship as a function of percentiles of years active, h-index, and number of publications. Results are from a model adjusting for percentiles of years active, h-index, and number of publications and allowing for an interaction term between the linear effect of h-index and gender. Results are adjusted for field of expertise through matching.

© 2019 Thomas EG et al. *JAMA Network Open.*

eTable 5. Results From Conditional Logistic Regression Model Including Interaction Term Between Gender and Number of Publications

Variable	Comparison	Odds Ratio	95%CI
Gender	Female vs. male, for authors at lowest percentile of number of publications	1.00	(0.94, 1.06)
Gender x number of publications percentile [a]	Female vs. male/10-point increase	0.96	(0.95, 0.97)
Years active percentile [b]		See eFigure 6	
H-index percentile [b]		See eFigure 6	
Number of publications percentile [b]		See eFigure 6	

[a] The interaction between gender and number of publications is visualized in eFigures 6(a) and 7(c).
[b] Years since first publication, h-index, and number of publications were included in models as percentiles and were adjusted for using natural cubic splines to allow for non-linear effects. The odds ratio as a function of these variables is displayed in eFigure 6, since coefficients for spline terms are not interpretable. Numeric results are available from the authors upon request, or by accessing full results at github.com/emgthomas/gender_and_invited_commentaries.

© 2019 Thomas EG et al. *JAMA Network Open.*

eFigure 6. Results From Conditional Logistic Regression Model Including Interaction Term Between Gender and Number of Publications

(a) Odds ratio as a function of number of publications by gender

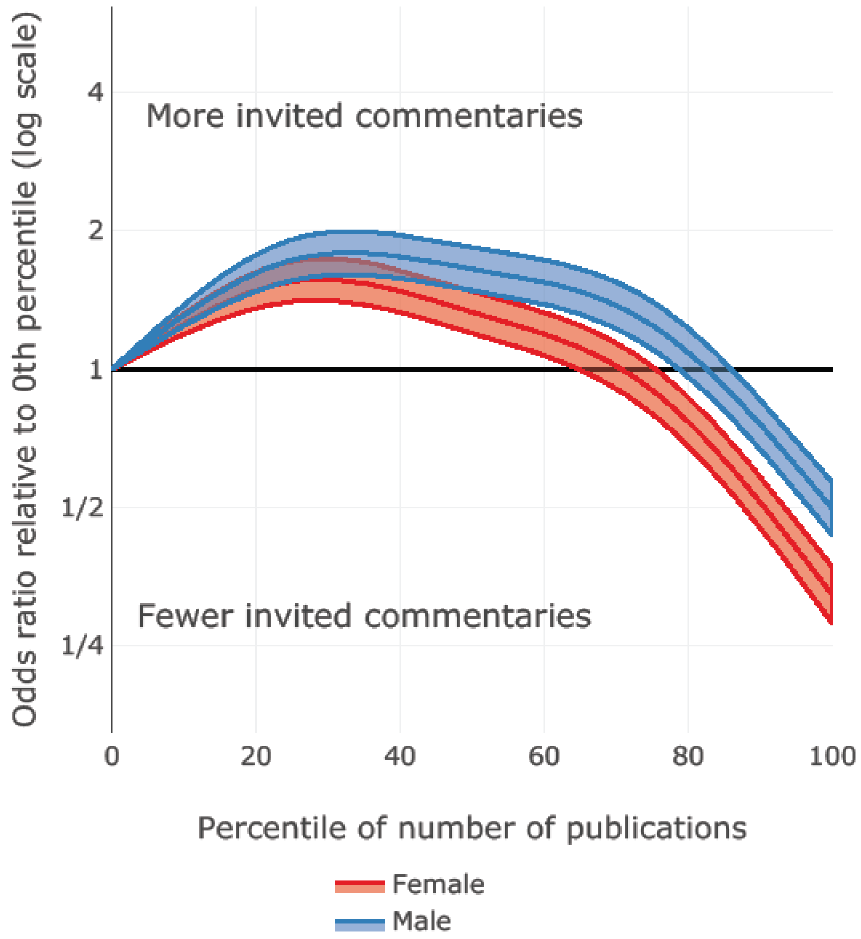

© 2019 Thomas EG et al. *JAMA Network Open.*

(b) Odds ratio as a function of years active and h-index

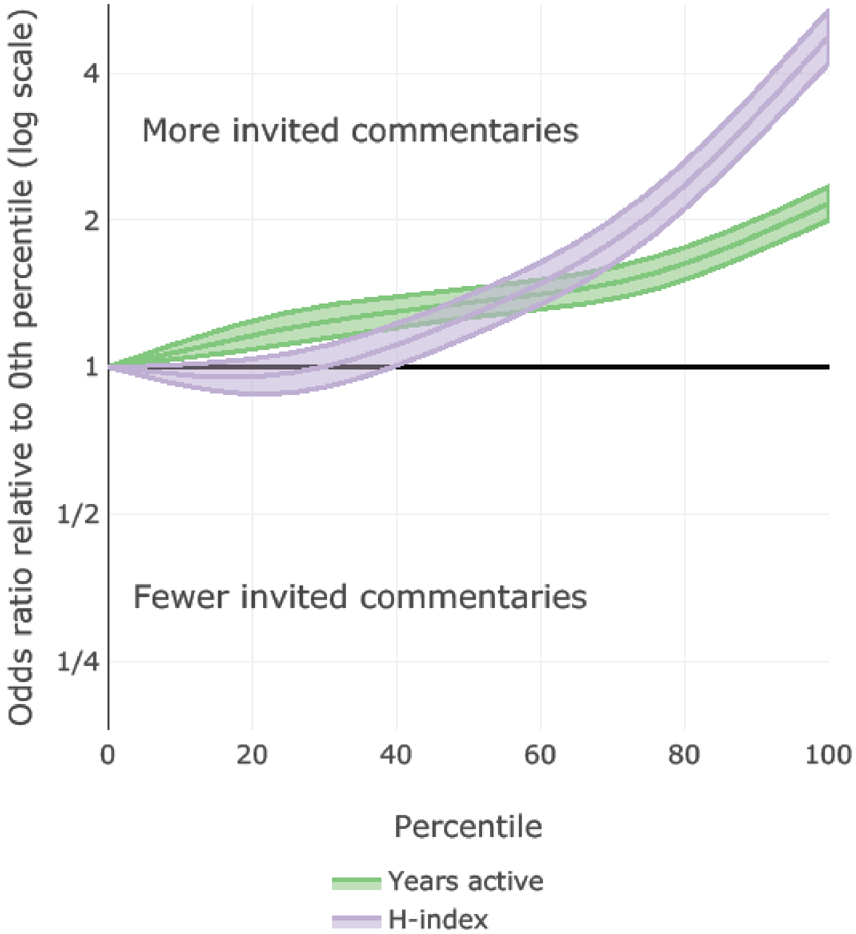

Caption: The figure shows the odds ratio for invited commentary authorship as a function of percentiles of years active, h-index, and number of publications. Results are from a model adjusting for percentiles of years active, h-index, and number of publications and allowing for an interaction term between the linear effect of number of publications and gender. Results are adjusted for field of expertise through matching.

© 2019 Thomas EG et al. *JAMA Network Open.*

eFigure 7. Association of Gender With Authoring an Invited Commentary

(a) Odds ratio by years active

Years Active	Percentile of Years Active	OR (95%CI)
8	10th	0.90 (0.86,0.94)
14	30th	0.84 (0.81,0.87)
19	50th	0.79 (0.77,0.81)
27	70th	0.73 (0.71,0.76)
38	90th	0.69 (0.66,0.72)

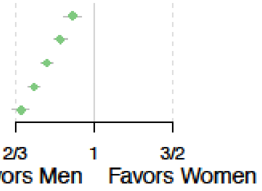

(b) Odds ratio by h-index

H–Index	Percentile of H–Index	OR (95%CI)
7	10th	0.93 (0.89,0.98)
13	30th	0.87 (0.84,0.90)
20	50th	0.80 (0.78,0.82)
30	70th	0.74 (0.72,0.76)
51	90th	0.69 (0.66,0.72)

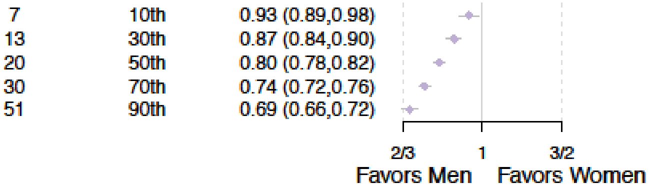

(c) Odds ratio by years active

Number of Publications	Percentile of Number of Publications	OR (95%CI)
20	10th	0.96 (0.91,1.01)
41	30th	0.88 (0.85,0.91)
71	50th	0.80 (0.78,0.83)
123	70th	0.74 (0.72,0.76)
252	90th	0.68 (0.65,0.70)

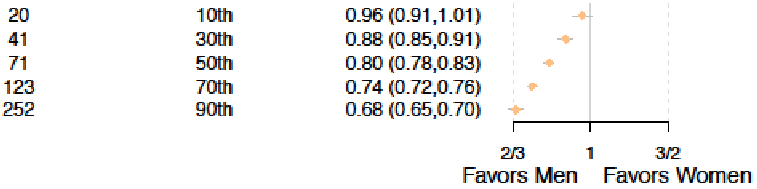

Caption: Subfigures (a), (b), and (c) show the odds ratio of invited commentary authorship for women compared to men, esimated from a model including a single interaction term between gender and one of years active percentile, h-index percentile or number of publications percentile, respectively. Each model also controls for the other variables and is adjusted for field of scientific expertise through matching.

© 2019 Thomas EG et al. *JAMA Network Open.*

eAppendix 3. Estimated Journal-Specific Odds Ratios vs Journal CiteScore

An interactive figure and corresponding data representing the estimated journal-specific odds ratios vs journal CiteScore is available at available at:
https://emgthomas.shinyapps.io/gender_and_invited_commentaries/

© 2019 Thomas EG et al. *JAMA Network Open.*

eTable 6. Association of Gender With Invited Commentary Authorship Estimated via 2-Stage Random Effects Meta-Analysis

**Model**	**Variable**	**Comparison**	**Odds Ratio**	**95%CI**	I^2	p_{het}
Adjusted for field of expertise only						
	Gender	Female vs. male	0.77	(0.74, 0.79)	15.6%	0.2718
Fully adjusted [a]						
	Gender	Female vs. male	0.80	(0.77, 0.82)	5.9%	0.8081
Fully adjusted with interaction [a]						
	Gender	Female vs. male, for authors at lowest percentile of years active	0.97	(0.91, 1.04)	0.3%	0.9895
	Gender x years active percentile	Female vs. male or 10-point increase	0.97	(0.96, 0.98)	2.6%	0.8562

[a] Years since first publication, h-index, and number of publications were included in adjusted models as percentiles using natural cubic splines to allow for non-linear effects.

Caption: The table shows results obtained using random effects meta-analysis to pool journal-specific effect estimates. This analysis included 1,410 journals with sufficient data to obtain journal-specific effects. I^2 is an estimate of the percent of total between-journal variation in effect size that can be attributed to true between journal heterogeneity, rather than sampling variability. p_{het} is the p-value from a test of the null hypothesis of no between-journal heterogeneity in true effect sizes.

© 2019 Thomas EG et al. *JAMA Network Open.*

eFigure 8. 2-Stage Meta-Analysis Results by Journal Topic

Topic	ASJC	Journals	Cases	Model 1 OR (95%CI)	Model 2 OR (95%CI)
General	1000	3	429	0.83 (0.64,1.07)	0.96 (0.73,1.27)
Medicine (all)	2700	50	2756	0.71 (0.59,0.87)	0.76 (0.64,0.89)
Medicine (miscellaneous)	2701	50	1014	0.95 (0.78,1.16)	0.90 (0.74,1.10)
Anatomy	2702	9	57	1.10 (0.59,2.03)	1.09 (0.43,2.74)
Anesthesiology and Pain Medicine	2703	36	1121	0.81 (0.67,0.97)	0.79 (0.65,0.95)
Biochemistry, Medical	2704	15	303	0.52 (0.37,0.72)	0.50 (0.35,0.74)
Cardiology and Cardiovascular Medicine	2705	110	7368	0.76 (0.69,0.85)	0.83 (0.74,0.92)
Critical Care and Intensive Care Medicine	2706	26	1494	0.75 (0.62,0.90)	0.76 (0.64,0.90)
Complementary and Alternative Medicine	2707	7	53	0.77 (0.40,1.48)	0.58 (0.25,1.34)
Dermatology	2708	24	716	0.75 (0.63,0.90)	0.77 (0.63,0.96)
Embryology	2710	4	35	0.50 (0.21,1.16)	N/A
Emergency Medicine	2711	16	392	0.64 (0.47,0.86)	0.67 (0.48,0.92)
Endocrinology, Diabetes, and Metabolism	2712	54	1448	0.77 (0.66,0.90)	0.78 (0.67,0.90)
Epidemiology	2713	45	1180	0.81 (0.71,0.92)	0.83 (0.71,0.96)
Family Practice	2714	10	155	0.61 (0.41,0.92)	0.59 (0.37,0.93)
Gastroenterology	2715	35	1308	0.68 (0.55,0.82)	0.73 (0.60,0.87)
Genetics (clinical)	2716	34	535	0.82 (0.67,1.02)	0.81 (0.63,1.03)
Geriatrics and Gerontology	2717	28	352	0.80 (0.62,1.03)	0.72 (0.53,0.96)
Health Informatics	2718	13	196	1.02 (0.64,1.62)	0.83 (0.46,1.50)
Health Policy	2719	66	1269	0.81 (0.70,0.94)	0.70 (0.59,0.83)
Hematology	2720	42	1768	0.72 (0.62,0.83)	0.73 (0.64,0.84)
Hepatology	2721	19	866	0.75 (0.56,1.02)	0.78 (0.60,1.01)
Histology	2722	19	159	0.77 (0.52,1.15)	0.74 (0.40,1.37)
Immunology and Allergy	2723	59	1906	0.74 (0.65,0.84)	0.81 (0.71,0.93)
Internal Medicine	2724	34	2084	0.69 (0.58,0.82)	0.73 (0.63,0.85)
Infectious Diseases	2725	81	2508	0.82 (0.74,0.92)	0.86 (0.77,0.95)
Microbiology (medical)	2726	29	736	0.68 (0.57,0.81)	0.75 (0.61,0.91)
Nephrology	2727	16	986	0.87 (0.69,1.10)	0.88 (0.73,1.06)
Clinical Neurology	2728	103	3101	0.71 (0.62,0.80)	0.72 (0.64,0.81)
Obstetrics and Gynecology	2729	43	795	0.74 (0.59,0.91)	0.77 (0.60,0.98)
Oncology	2730	89	3231	0.83 (0.75,0.93)	0.91 (0.82,1.00)
Ophthalmology	2731	33	499	0.91 (0.70,1.17)	0.90 (0.67,1.21)
Orthopedics and Sports Medicine	2732	44	740	0.90 (0.71,1.15)	0.72 (0.56,0.93)
Otorhinolaryngology	2733	15	173	0.83 (0.56,1.25)	0.82 (0.50,1.36)
Pathology and Forensic Medicine	2734	38	363	0.69 (0.53,0.90)	0.63 (0.45,0.89)
Pediatrics, Perinatology, and Child Health	2735	83	1993	0.77 (0.68,0.88)	0.82 (0.72,0.93)
Pharmacology (medical)	2736	56	1289	0.93 (0.79,1.09)	0.92 (0.79,1.06)
Physiology (medical)	2737	36	1619	0.70 (0.58,0.86)	0.79 (0.62,0.99)
Psychiatry and Mental Health	2738	136	2448	0.68 (0.61,0.75)	0.72 (0.65,0.81)
Public, Environmental, and Occupational Health	2739	121	1759	0.91 (0.81,1.03)	0.95 (0.84,1.08)
Pulmonary and Respiratory Medicine	2740	50	2681	0.65 (0.58,0.73)	0.72 (0.63,0.81)
Radiology Nuclear Medicine and Imaging	2741	73	1788	0.79 (0.69,0.90)	0.84 (0.72,0.98)
Rehabilitation	2742	22	232	1.05 (0.77,1.43)	1.08 (0.75,1.57)
Reproductive Medicine	2743	18	344	0.80 (0.55,1.16)	0.80 (0.49,1.28)
Rheumatology	2745	19	590	0.88 (0.72,1.06)	0.86 (0.70,1.06)
Surgery	2746	70	2489	0.85 (0.72,0.99)	0.92 (0.78,1.09)
Transplantation	2747	15	901	0.86 (0.72,1.03)	0.88 (0.73,1.06)
Urology	2748	21	1073	0.57 (0.46,0.71)	0.63 (0.48,0.82)
**All**		**2556**	**46235**	**0.77 (0.74,0.79)**	**0.80 (0.77,0.82)**

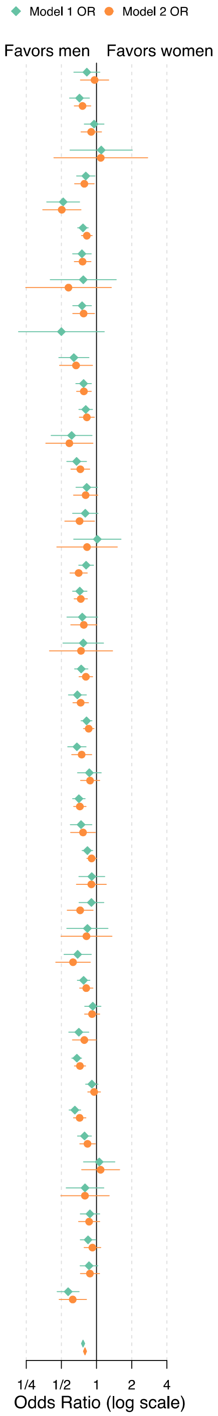

Caption: The figure shows results sub-group analyses for journals by topic, as denoted by All Science Journal Classification (ASJC) codes. Journals may have multiple ASJC codes; thus, the topics are overlapping with respect to journals included. Unadjusted models control for authors' fields of expertise through matching. Adjusted models further control for years active percentile, h-index percentile, and total number of publications percentile. Estimates that could not be obtained due to small sample sizes are shown as N/A.

© 2019 Thomas EG et al. *JAMA Network Open.*

eFigure 9. Meta-Regression of Journal-Specific Effect Sizes Against CiteScore

(a) Odds ratio adjusted for field of expertise

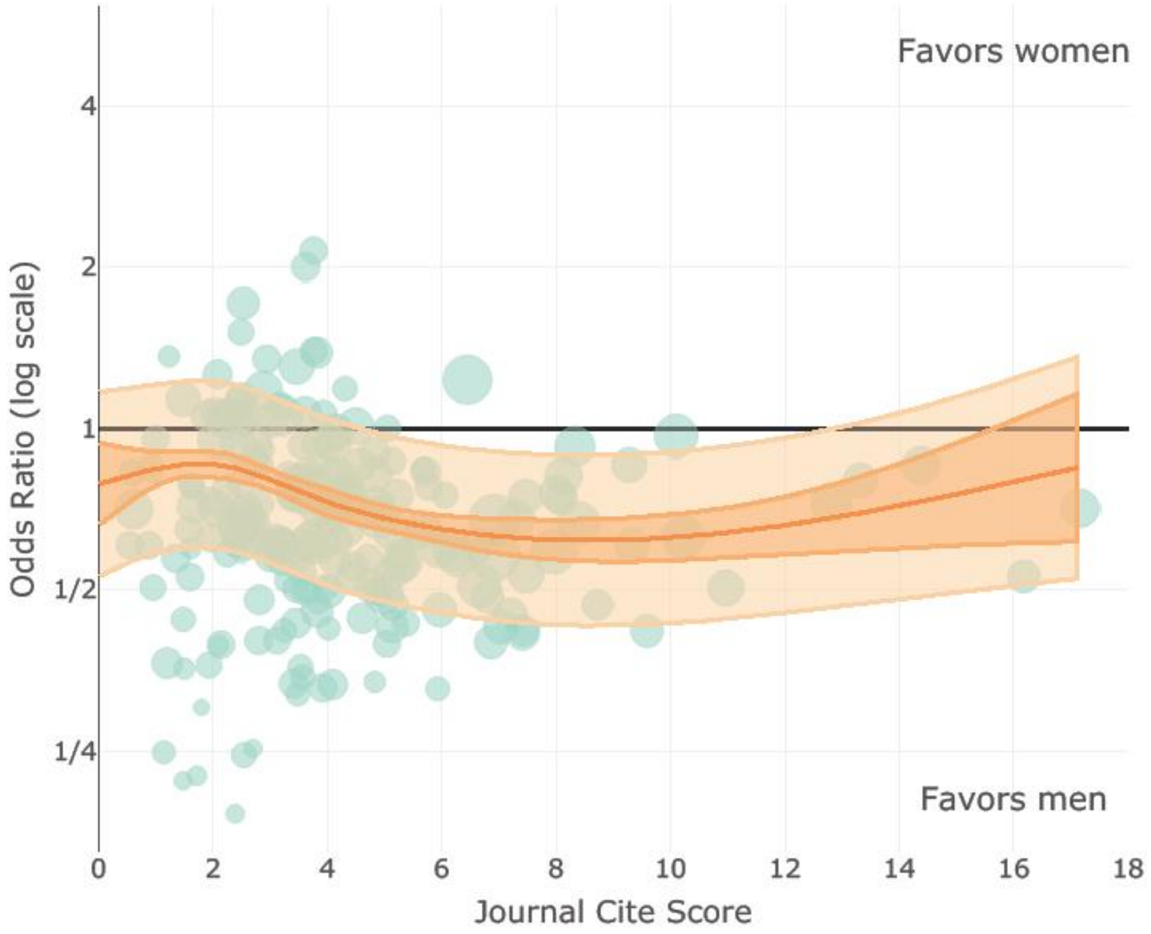

© 2019 Thomas EG et al. *JAMA Network Open.*

(b) Odds ratio adjusted for field of expertise, years active, h-index and number of publications

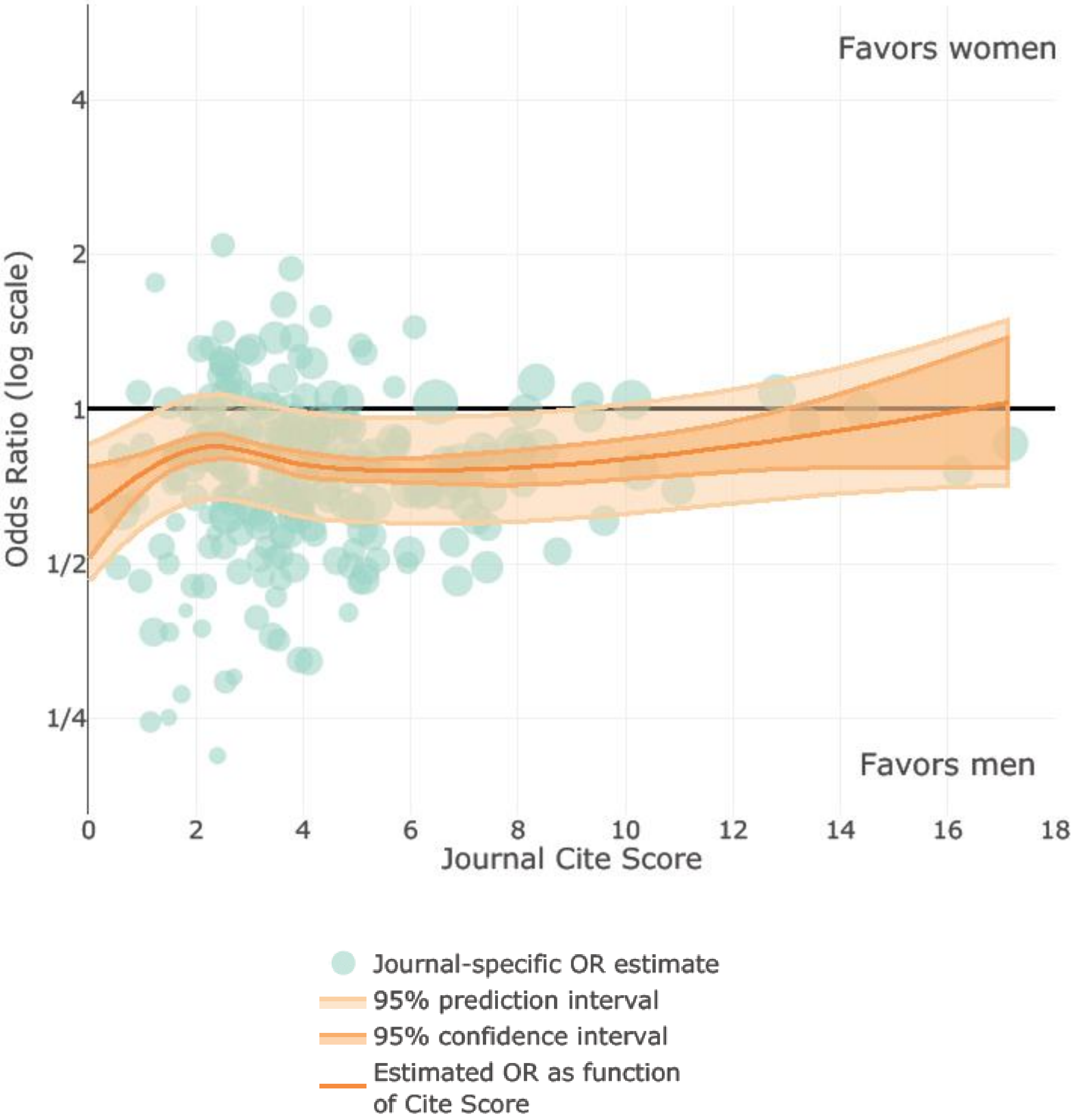

Caption: The line, confidence interval and prediction interval represent the predicted odds ratio for women vs. men of authoring invited commentaries as a function of journal Cite Score, as estimated via meta-regression. Each circle represents the odds ratio estimated for a single journal. Circle diameter is inversely proportional to the standard error of the log odds ratio estimate. For clarity, only journals with more than 50 matched sets are shown. Unadjusted models control for authors' fields of expertise through matching. Adjusted models further control for years active percentile, h-index percentile, and total number of publications percentile.

© 2019 Thomas EG et al. *JAMA Network Open.*

eTable 7. Association of Gender With Invited Commentary Authorship Accounting for Missing Gender Data via Multiple Imputation

**Model**	**Variable**	**Comparison**	**Odds Ratio**	**95%CI**
Adjusted for field of expertise only				
	Gender	Female vs. male	0.683	(0.668, 0.698)
Fully adjusted [a]				
	Gender	Female vs. male	0.760	(0.743, 0.778)
Fully adjusted with interaction [a]				
	Gender	Female vs. male, for authors with median years since first publication	0.872	(0.828, 0.917)
	Gender x years active percentile	Female vs. male/10-point increase	0.975	(0.967, 0.983)

[a] Years since first publication, h-index, and number of publications were included in adjusted models as percentiles using natural cubic splines to allow for non-linear effects. Odds ratios and standard errors were combined over ten imputed datasets using Rubin's rules.

© 2019 Thomas EG et al. *JAMA Network Open.*

eTable 8. Association of Gender on Invited Commentary Authorship After Excluding Duplicates for Case Authors Who Appear in Multiple Journals, Excluding Articles That May Be Replies to Other Articles, and Keeping Only Top 2 Controls Per Case

**Model**	**Variable**	**Comparison**	**Odds Ratio**	**95%CI**
Adjusted for field of expertise only				
	Gender	Female vs. male	0.76	(0.73, 0.78)
Fully adjusted [a]				
	Gender	Female vs. male	0.79	(0.76, 0.82)
Fully adjusted with interaction [a]				
	Gender	Female vs. male, for authors with median years since first publication	0.94	(0.88, 1.01)
	Gender x years active percentile	Female vs. male/10-point increase	0.97	(0.95, 0.98)

[a] Years since first publication, h-index, and number of publications were included in adjusted models as percentiles using natural cubic splines to allow for non-linear effects. All results are adjusted for field of scientific expertise through matching.

© 2019 Thomas EG et al. *JAMA Network Open.*

eReferences

1. genderize.io. Genderize.io | Determine the gender of a first name. https://genderize.io/. Accessed 3/28, 2019.
2. Robertson S, Zaragoza H, others. The probabilistic relevance framework: BM25 and beyond. *Foundations and Trends in Information Retrieval.* 2009;3(4):333-389.
3. Robertson SE, Walker S. Some simple effective approximations to the 2-poisson model for probabilistic weighted retrieval. Paper presented at: SIGIR'941994.
4. Lin S, Hong W, Wang D, Li T. A survey on expert finding techniques. *Journal of Intelligent Information Systems.* 2017;49(2):255-279.
5. Robertson SE, Walker S, Beaulieu M. Experimentation as a way of life: Okapi at TREC. *Information processing & management.* 2000;36(1):95-108.
6. Zhai C. Statistical language models for information retrieval. *Synthesis Lectures on Human Language Technologies.* 2008;1(1):1-141.
7. Trotman A, Puurula A, Burgess B. Improvements to BM25 and language models examined. Paper presented at: Proceedings of the 2014 Australasian Document Computing Symposium2014.
8. Kang N, Doornenbal MA, Schijvenaars RJ. Elsevier journal finder: recommending journals for your paper. Paper presented at: Proceedings of the 9th ACM Conference on Recommender Systems2015.
9. Schuemie MJ, Kors JA. Jane: suggesting journals, finding experts. *Bioinformatics.* 2008;24(5):727-728.
10. Simmonds MC, Higginsa JP, Stewartb LA, Tierneyb JF, Clarke MJ, Thompson SG. Meta-analysis of individual patient data from randomized trials: a review of methods used in practice. *Clinical Trials.* 2005;2(3):209-217.
11. La Gamba F, Corrao G, Romio S, et al. Combining evidence from multiple electronic health care databases: performances of one-stage and two-stage meta-analysis in matched case-control studies. *Pharmacoepidemiology and Drug Safety.* 2017;26(10):1213-1219.
12. Santamaría L, Mihaljević H. Comparison and benchmark of name-to-gender inference services. *PeerJ Computer Science.* 2018;4:e156.
13. Larivière V, Ni C, Gingras Y, Cronin B, Sugimoto CR. Bibliometrics: Global gender disparities in science. *Nature News.* 2013;504(7479):211.
14. Elsevier. *Gender in the global research landscape.* Elsevier.
15. Anderson GB, Barnes EA, Bell ML, Dominici F. The future of climate epidemiology: opportunities for advancing health research in the context of climate change. *American Journal of Epidemiology.* 2019;188(5):866-872.

© 2019 Thomas EG et al. *JAMA Network Open.*
